# Sodium-glucose transporters SGLT1 and SGLT2 in equine renal, hepatic and pancreatic tissue

**DOI:** 10.1186/s12917-025-05173-1

**Published:** 2025-12-17

**Authors:** Camilla Anger-Håål, Cathrine T. Fjordbakk, Carl Ekstrand, Fredrik S. Skedsmo, Runa Rørtveit

**Affiliations:** 1 Department of Preclinical Sciences and Pathology, Faculty of Veterinary Medicine, Norwegian University of Life Sciences, Ås, Norway; 2 Department of Companion Animal Clinical Sciences, Faculty of Veterinary Medicine, Norwegian University of Life Sciences, Ås, Norway; 3 Department of Animal Biosciences, Faculty of Veterinary Medicine and Animal Science, Swedish University of Agricultural Sciences, Uppsala, Sweden

**Keywords:** Equine metabolic syndrome, Sodium-glucose linked transporters, Sodium-dependent glucose transporter, SGLT2-inhibitors, Hyperinsulinemia-associated laminitis, Canagliflozin

## Abstract

**Background:**

Insulin dysregulation is a hallmark of equine metabolic syndrome (EMS), and in recent years, pharmacological treatment with sodium-dependent glucose transporter 2 inhibitors (SGLT2i) have shown promise in reducing the risk of hyperinsulinemia-associated laminitis in horses diagnosed with EMS. In humans and laboratory animals, this transporter protein is responsible for the majority of renal tubular glucose reabsorption, however, the presence of this and the related sodium-dependent glucose transporter 1 (SGLT1) have not yet been studied in equine kidneys. Additionally, studies in humans and laboratory animals have documented the presence of SGLT1 and SGLT2 also in hepatic and pancreatic tissue, which may explain extra-renal treatment effects of SGLT2i. Since the specificity towards SGLT2 and SGLT1 differ between the various SGLT2i drugs currently in use in horses, investigating SGLT2 and SGLT1 protein expression in equine tissues may help understanding potential differences in treatment effect and/or side effect profile between substances. The objective of this study was therefore to evaluate the presence of SGLT2 and SGLT1 in equine kidneys, liver and pancreas.

**Results:**

Tissue samples from ten healthy Norwegian/Swedish Coldblood Trotters were collected. Using immunohistochemistry with antibodies raised against human SGLT1 and SGLT2, a strong SGLT2 antibody signal was present in the apical membranes of epithelial cells in the cortical labyrinth of the kidney, while SGLT1 positive cells were predominantly found in medullary rays. Both of these results concur with those in humans and rats. Using electron microscopy, ultrastructural localisation of positive SGLT2 antibody signal was confirmed to the microvilli of tubular epithelial cells. Positive SGLT2 signal was also detected in periportal hepatocytes and in cells within islet of Langerhans in the endocrine pancreas. Positive SGLT1 signal was seen in cholangiocytes in the portal areas of the liver, and in Kuppfer cells.

**Conclusions:**

The present study confirms presence of SGLT2 and SGLT1 in the equine kidney, localized to the proximal tubule. Also, presence of SGLT2 in the liver and pancreas, suggest that SGLT2i may have both renal and extrarenal effects.

**Supplementary Information:**

The online version contains supplementary material available at 10.1186/s12917-025-05173-1.

## Background

Equine metabolic syndrome (EMS) is a set of risk factors associated with increased risk of hyperinsulinemia-associated laminitis (HAL) and other co-morbidities such as regional or generalized adiposity, hypertension and altered lipid metabolism [1]. Insulin dysregulation (ID) is a hallmark feature of EMS [2], encompassing fasting hyperinsulinemia, post-prandial hyperinsulinemia and peripheral tissue insulin resistance [2]. The prevalence of EMS is high in genetically predisposed breeds, especially ponies, cobs, gaited horses, Warmbloods, Spanish breeds, Morgans and Arabians [3, 4] and has been reported to account for 89% of all laminitic cases presented to an equine hospital [5]. Although experimentally induced hyperinsulinemia consistently evokes laminitis in healthy horses [6, 7], the exact pathophysiology of HAL has yet to be elucidated.

Treatment of EMS is mainly focused on lifestyle alterations, such as dietary changes to encourage weight loss as well as increased physical activity [2]. However, increased activity levels cannot be implemented in periods of acute or chronic lameness. Few options for pharmacological treatment exist, and metformin (a human anti-hyperglycemic drug) and levothyroxine (synthetic thyroid hormone) have shown variable results [8–15]. Since 2018, there has been an increasing interest in the human diabetes medication group sodium-dependent glucose transporter 2 (SGLT2)-inhibitors (SGLT2i) as a potential treatment for EMS, as this class of drugs have been shown to reduce hyperinsulinemia and increase insulin sensitivity in horses [16–21]. The proposed mechanism of action of the SGLT2i is by blocking glucose reabsorption in the proximal renal tubules, leading to increased urinary glucose excretion, as previously shown in mice [22].

Studies performed in rodents and humans reveal that SGLT2 is predominately situated in the apical membrane of epithelial cells in the first part of proximal renal tubules, however, SGLT2 proteins and mRNA from the SGLT2 gene Solute Carrier Family 5 Member 2 (*SLC5A2*) have also been found in other tissues such as the liver, intestines, spleen, heart and brain [23–27]. The structurally similar sodium-dependent glucose transporter 1 (SGLT1) is most abundant in small intestine enterocytes and in the distal segment of the proximal renal tubules [28–34]. Both SGLT1 proteins and SLC5A1 mRNA are also found in many other tissues, such as the heart, testes, prostate, brain, spinal cord, colon, trachea, lung, pancreas, uterus and liver [29].

The different SGLT2i differ in their selectivity of SGLT2 versus SGLT1 inhibition; for instance, canagliflozin, which is the most commonly used SGLT2i drug in the Nordic veterinary market, demonstrates relatively low selectivity for SGLT2 [35]. Whereas presence of intestinal SGLT1 has been demonstrated in horses [36, 37], there are no detailed descriptions on presence and location of SGLT2 in equine tissues. Therefore, the aim of this study was to characterize the distribution of SGLT1 and SGLT2 in kidney, pancreas and liver in systemically healthy horses in order to increase our knowledge on the potential clinical consequences of SGLT2i treatment in horses.

## Materials and methods

### Animals and tissue sampling

A convenience sample of organs harvested from 10 privately owned adult Norwegian/Swedish Coldblood Trotters (NSCT) presenting for elective euthanasia at the Norwegian University of Life Sciences was collected; complete information on included horses is provided in Supplementary Table 1. Horses were only included after obtaining a signed consent form by their owners or owner representatives. Reasons given for euthanasia included lameness, infertility in brood mares, or were undisclosed. Exclusion criteria were any signs of systemic disease apparent from stated medical history, clinical examination or macroscopical evaluation of organs at necropsy. Horses were stunned with captive bolt technique and immediately exsanguinated. Organs were sampled within 2 h of euthanasia. From each horse, samples were obtained from the left kidney, the left lobe of the pancreas and the left lateral lobe of the liver. Tissues were immediately placed in fixation media as described below. For light microscopy, formalin-fixed tissues were paraffin-embedded before 5 μm sections were placed on glass slides. Slides were deparaffinated, stained with hematoxylin and eosin and coverslips mounted using an automated stainer and coverslip machine (Tissue-Tek Prisma Plus and Tissue-Tek Glas g2). Light microscopy images were obtained and scanned using a digital scanner (Philips Pathology Scanner SG300).

The study was reviewed by the Ethical committee for approval of studies with animal patients, an ethical committee affiliated to the Norwegian University of Life Sciences. The ethical committee concluded that the study did not meet the criteria for studies that require application to or approval from any ethical committee.

### Sample preparation for immunohistochemistry

Immunohistochemistry was performed on formalin-fixed, paraffin-embedded tissue. Section 3 μm thick were mounted onto glass slides (Superfrost Plus^®^, Menzel Gläser) and stored at 4 °C before use. Slides were deparaffinized and heat-induced antigen retrieval performed in a pressure cooker (slides were heated to 110 °C, for 15 min) with a citrate-based buffer (DIVA Decloaker, DV2004, Biocare Medical). Endogenous peroxidase and proteins were blocked using hydrogen peroxide solution (Peroxidased 1, PX968, Biocare Medical) and a casein-based protein blocking agent (Background Sniper, BS966, Biocare Medical), respectively. Slides were incubated with primary antibodies raised in rabbits against human SGLT1 (SGLT-101AP, Fabgennix) or SGLT2 (PA5-34210, Invitrogen) overnight at 4 °C. Antibodies were diluted 1:1000 (SGLT2) and 1:1500 (SGLT1) in a PBS-based diluent (Da Vinci Green Diluent, Biocare Medical). Between steps, slides were repeatedly washed in phosphate-buffered saline (PBS). Incubation with a secondary horseradish peroxidase -conjugated antibody (MACH1 Universal HRP-polymer, MRH538, Biocare Medical) was done at room temperature for 30 min. Slides were subsequently incubated with a chromogen (AEC Romulin, RAEC810, Biocare Medical) for 6 min. After counterstaining with hematoxylin, slides were rinsed in deionized water before coverslips were mounted using an automated stainer and coverslip machine (Tissue-Tek Prisma Plus and Tissue-Tek Glas g2). Kidney tissue was used as a positive control for liver and pancreas tissues, and for negative control only antibody diluent was used in the first incubation step. Negative controls did not show any staining in any of the tissues. Results were validated with a second pair of commercial antibodies raised against different epitopes for SGLT1 (PA5-84237, Invitrogen) and SGLT2 (ab85626, Abcam) using the same protocol, in concentrations 1:5000 (SGLT1) and 1:10000 (SGLT2). For additional information on antibodies used, see Supplementary Table 2. Images for light microscopy were obtained using the digital scanner mentioned above. Interpretation of staining results were based on criteria defined in Supplementary image S1.

### Sample preparation for electron microscopy

Immunolabeling was performed as previously described with minor modifications [38]. Briefly, kidney tissue from two horses were fixed by immersion in 0.1% glutaraldehyde (GA) and 4% paraformaldehyde in PIPES (piperazine-N, N′-bis(2-ethanesulfonic acid)-buffer for 4 h, followed by washing with PIPES-buffer and subsequent storage in PIPES-buffer containing 1% PFA at 4 °C until time of analysis. Samples were embedded in 12% gelatine, cut into 1 mm^3^ cubes with a razor blade and infiltrated with 2.3 M sucrose overnight at 4 °C. Samples were subsequently mounted on sample pins (Electron Microscopy Sciences) and snap frozen in liquid nitrogen, trimmed and cut into 70 nm sections on an ultramicrotome (Leica EM UC6 with a FC7 cryo unit). The sections were picked up with a loop by using a solution consisting of 1.15 M sucrose and 1% methyl cellulose, and mounted on formvar and carbon-coated copper mesh grids. Immunolabeling was performed by first incubating the grids in PBS for 30 min at 37 °C, before blocking in steps with 0.1% glycine in PBS and 1% bovine serum albumin (BSA) in PBS, respectively. The grids were subsequently incubated for 30 min with anti-SGLT2 antibody from Invitrogen diluted 1:100 in 1% BSA, before washing in 0.1% BSA. Next, grids were incubated for 20 min with Protein-A-Gold (10 nm, Cell Microscopy Core, Utrecht) diluted 1:50 in PBS, before washing in PBS. Thereafter, the grids were incubated with 1% GA in PBS for 5 min, washed in deionized water and contrasted with 0.4% uranyl acetate in 2% methyl cellulose on ice for 5 min. The grids were allowed to air-dry and then examined in a Jeol transmission electron microscope (JEM 2100-Plus).

## Results

### SGLT1 and SGLT2 in the kidney

**Fig. 1:**
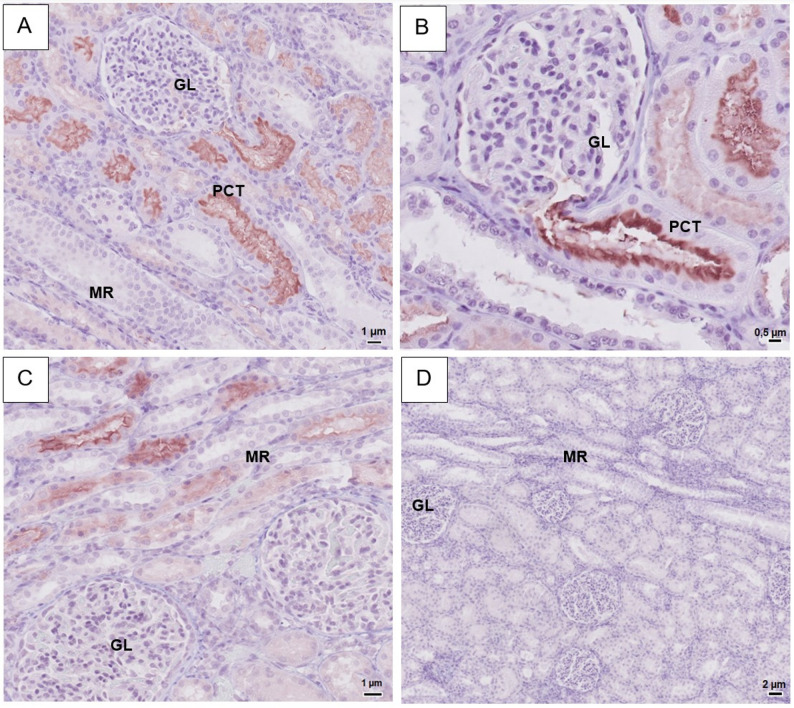
Representative light microscopy images of equine renal tissue immunohistochemistry using a SGLT1 or SGLT2-primary rabbit anti-human antibody. For more details on methods, see main text. **A**-**B**: IHC with SGLT2 (Invitrogen), strong staining visible in brush border of epithelial cells in tubules in the cortical labyrinth, strongest signal in most proximal part of proximal convoluted tubules (see figure B). **C**: IHC with SGLT1 (Fabgennix), strong signal apparent in brush border of epithelial cells in tubules within the medullary rays. **D**: Negative control – no staining visible. PCT = Proximal convoluted tubules, GL = glomeruli, MR = medullary rays

In the renal cortex, the Invitrogen SGLT2 antibody stained the brush border of the epithelial cells of the proximal convoluted tubules, while basolateral cell membranes showed no staining (Fig. 1A). Intensity of staining was highest in the proximal part of the proximal tubules (Fig. 1B). Glomeruli, blood vessels and distal tubules were unstained. In the cortical medullary rays, weak staining was seen in the brush border of proximal straight tubules. Collecting ducts were unstained.

A SGLT1 signal was seen in the brush border of epithelial cells of the proximal straight tubules of the cortical medullary rays with an increase in intensity towards the medulla (Fig. 1C), while a weak positive staining was seen in the brush border of epithelial cells of the proximal convoluted tubules. Glomeruli and collecting ducts showed no staining. Validation with the second set of antibodies showed similar staining patterns in renal tissue for both SGLT1 and SGLT2. However, the Invitrogen SGLT1 antibody showed a higher degree of unspecific background staining, necessitating high dilutions of the primary antibody and subsequently a milder intensity of the brush border signal compared to the results of the Fabgennix antibody. Negative controls remained wholly unstained (Fig. 1D).

### SGLT1 and SGLT2 in the liver

**Fig. 2:**
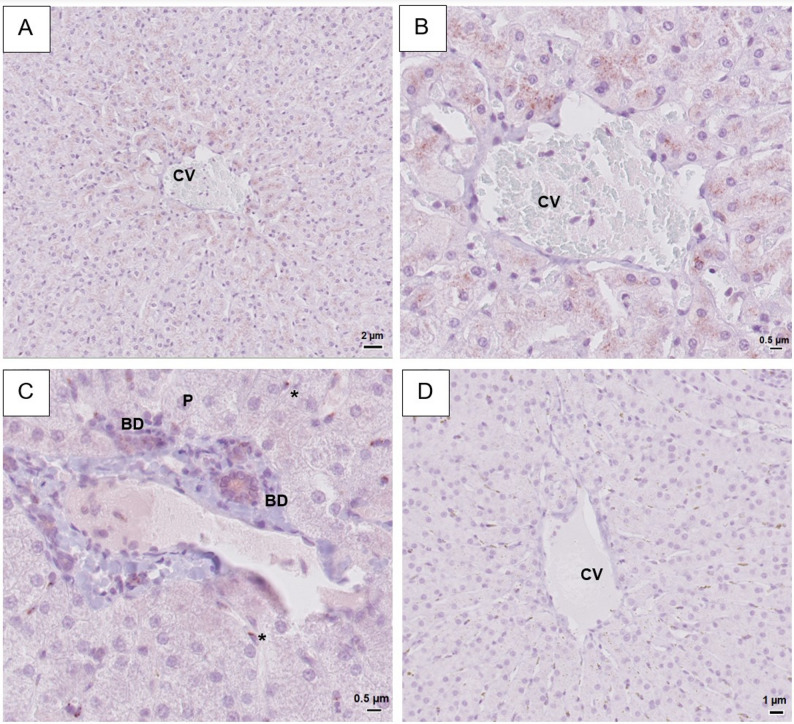
Representative light microscopy images of equine hepatic tissue. Immunohistochemistry was performed using a SGLT1 or SGLT2-primary rabbit anti-human antibody. For more details on methods, see main text. **A**, **B**: IHC with SGLT2 antibody (Invitrogen). Positive cytoplasmic signal in hepatocytes with signal increasing in strength towards the central vein. **C**: IHC with SGLT1 (Fabgennix). Positive signal in apical membrane of cholangiocytes in portal bile ducts. Several Kuppfer cells with granular cytoplasmic staining visible. **D**: Negative control. No positive staining. CV = central vein, P = portal area, BD = bile ducts, * = Kuppfer cells

In hepatic tissue stained with the Invitrogen SGLT2 antibody, a granular, cytoplasmic signal was seen in hepatocytes, with the strongest signal seen in cells closest to the central vein (Fig. 2A-B). The second SGLT2 antibody (Abcam) produced a weaker, diffuse cytoplasmic stain in both hepatocytes and Kuppfer cells close to centrolobular and periportal areas (Supplementary image S4D-F). For both antibodies, cholangiocytes and blood vessels remained unstained.

SGLT1 antibody (Fabgennix) showed weak but distinct staining on the apical membrane of cholangiocytes in the portal area (Fig. 2C). A granular cytoplasmic staining of Kuppfer cells was seen in all horses, but frequency varied between 10 and 40% positive Kuppfer cells (Fig. 2C). Blood vessels were unstained. The second SGLT1 antibody (Invitrogen) produced no signal in hepatic tissue, either in cholangiocytes or in Kuppfer cells (Supplementary image S5D-F). Negative controls remained wholly unstained (Fig. 2D).

Intracellular brown granular pigment was apparent in hepatic tissue from five horses. In two horses, granules were only apparent in Kuppfer cells while in three horses both hepatocytes and Kuppfer cells contained granules. This finding was also present in negative controls and H&E-stained sections and was interpreted as hemosiderin deposits (Supplementary image S6A).

### SGLT1 and SGLT2 in the pancreas

**Fig. 3:**
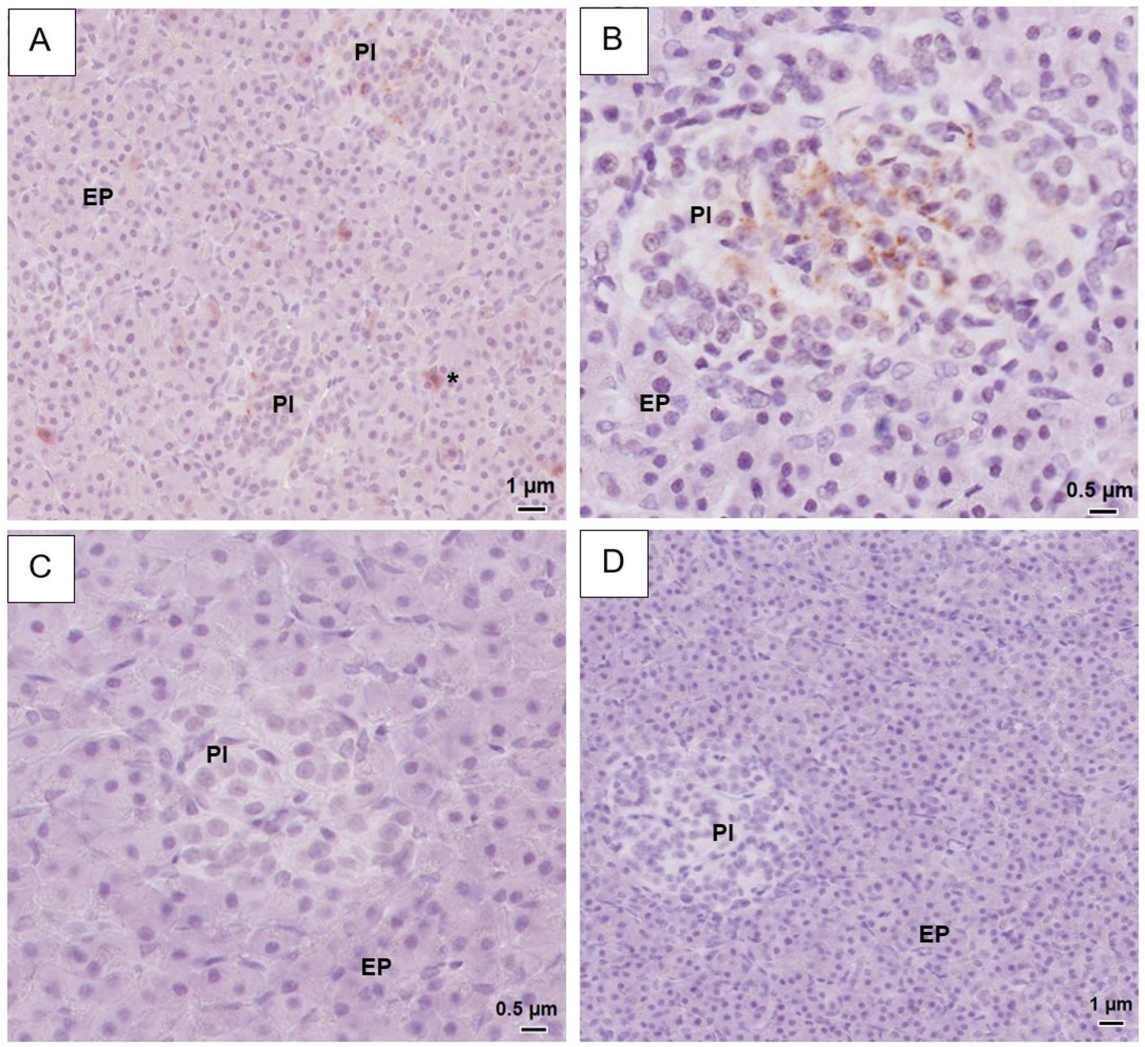
Representative light microscopy images of equine endocrine pancreatic tissue. Immunohistochemistry was performed using a SGLT1 or SGLT2-primary rabbit anti-human antibody. For more details on methods, see main text. **A**-**B**: IHC with SGLT2 (Invitrogen). Within the pancreatic islets, many cells with cytoplasmic fine granular staining are visualised, localised towards the middle of the islets. In exocrine pancreatic tissue, cytoplasmic staining of individual acinar cells is seen in A. **C**: IHC with SGLT1 (Fabgennix). No staining visible. **D**: Negative control. No staining visible. PI = pancreatic islets, EP = exocrine pancreas. * = positive acinar cells

In the pancreatic tissue, the two SGLT2 antibodies showed different staining patterns. The SGLT2 antibody from Invitrogen showed a positive fine granular staining of the pancreatic islets’ cells (Fig. 3A-B). Positive cells were localized towards the centre of the pancreatic islets (Fig. 3B). The intensity of this staining varied between the horses and was characterized as strong in five of the horses, and weak in the remaining five. In the exocrine pancreatic tissue, positive staining of the cytoplasm of individual acinar cells was observed in all horses (Fig. 3A). However, the number of such positively staining cells varied between individuals. In contrast, the Abcam antibody showed no positive staining of either endocrine or exocrine tissue (Supplementary image S4G-I). Neither one of the two SGLT1 antibodies showed a positive staining of the exocrine or endocrine pancreatic tissue (Fig. 3C, Supplementary image S5G-I). Negative controls remained unstained (Fig. 3D).

In three horses, a small amount of intracellular brown pigment was observed in individual exocrine pancreatic acinar cells (Supplementary image S6B) and was apparent both in H&E stained tissue and in negative control tissue. The appearance was similar to hemosiderin deposits observed in liver cells, and the individuals with these pancreatic findings also had the highest presence of hemosiderin in the liver.

### Ultrastructural location of SGLT2 in proximal tubular cells

**Fig. 4:**
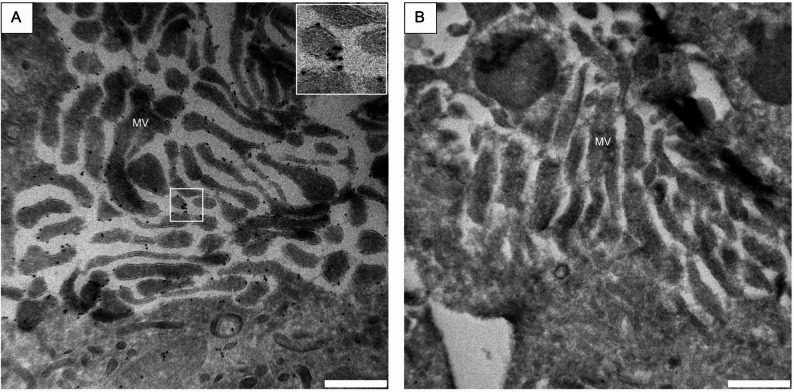
Transmission electron microscopy images of epithelial cells from proximal tubules of equine kidneys. Immunolabeling was performed using a SGLT2-antibody from Invitrogen with Protein-A-Gold with 10 nm gold particles. For more details on methods, see main text. **A**: Immunolabeling performed with SGLT2 antibody in concentration 1:100. A high density of gold particles can be seen bound to microvillar cell membranes, with lower amount of gold protein apparent in mitochondria and cytoplasm. Insert: Higher magnification of gold particles bound to microvillar membrane. **B**: Negative control – no specific binding of gold particles is visible. MV = microvilli. Scale bar = 500 nm

Ultrathin sections immunolabeled with SGLT2 antibody and Protein A Gold 10 nm particles showed a strong signal in the microvilli of the proximal tubular brush border (see Fig. 4A-B). Labelling was mostly present on the extracellular side of the membrane. Cell nucleus staining was very low and was deemed non-specific (data not shown). In high antibody concentration (1:100), some labelling of vesicles and mitochondria occurred (Fig. 4A), while in low antibody concentration (1:1000) cytoplasm and nuclei showed no to very low signal while labelling of microvilli was maintained (data not shown). No gold particles were visible in either microvilli, cell cytoplasm or organelles in negative controls (Fig. 4B).

## Discussion

In the kidney, the strongest SGLT2 staining was found in the renal cortical labyrinth, while SGLT1 showed higher staining intensity in medullary rays. This suggests that the equine SGLT1 and SGLT2 follow similar distribution patterns as described in humans [29] and rats [32], where protein expression of SGLT2 dominates in S1-S2 segments of the proximal tubules (in *pars convoluta)* while SGLT1 is predominantly found in S3 segments (in *pars recta*). Species differences in the renal distribution of these transport proteins do however exist, as the murine SGLT1 protein expression was stronger in the S2 than in the S3 segment [28]. Using immunogold labelling and transmission electron microscopy (TEM), the localisation of SGLT2 in the apical membrane of epithelial cells was confirmed. Both set of antibodies showed weak staining of SGLT2 in proximal straight tubules in medullary rays and of SGLT1 in proximal convoluted tubules in the cortical labyrinth, likely representing non-specific staining or possibly a cross-staining of the SGLT1 or 2 antibodies to SGLT2 or SGLT1 transporters, respectively. Although the antibodies had low overlap between immunogen sequences (see Supplementary Table 2), structural similarities between SGLT1, SGLT2 or other SGLT proteins might cause cross-reactivity in tissues where different SGLTs are present.

In other studied mammalian species, SGLT2 is responsible for 80–90% of total renal glucose reabsorption, whereas the contribution of SGLT1 is only 10–20% [39]. Our findings indicate that this likely is the case for equine renal glucose homeostasis also, and pharmacological inhibition of SGLT2 will therefore significantly decrease the renal reabsorption of glucose. However, while studies in humans and laboratory animals show that renal glucose reabsorption indeed is lower when SGLT2 are blocked or missing, a far lower amount of glucose is excreted than expected (38). This is most likely due to an overcapacity of glucose transport in the proximal tubules, and might also reflect an upregulation of SGLT1 transporters in response to increased glucose in the renal filtrate, as seen in humans with type 2 diabetes [40]. For that reason, choosing a less selective SGLT2-inhibitor which also partly inhibit SGLT1 might be beneficial for treatment in horses, as this could in part counteract the effects of this upregulation. However, relative affinity for different SGLT2-inhibitors have not yet been studied in horses. Research into glucose excretion changes over time in relation to treatment with different SGLT2i would elucidate this further.

In equine liver tissue, the Fabgennix SGLT1 antibody produced a positive signal in the apical membrane of cholangiocytes in the portal area bile ducts. Presence of SGLT1 in cholangiocytes has been demonstrated by protein expression studies in mice [28], humans [29] and rats [32]. This suggests the presence of a system for the reabsorption of glucose from bile. Glucose concentration in bile has been found to be low or virtually absent in rats and humans, respectively [41]. The same study saw an increase in bile glucose during high glucose intravenous infusion, indicative of a saturable transport system similar to the glucose transport occurring in renal tubules. Bile glucose concentration were shown to increase during studies in which rats were infused with non-selective SGLT-inhibitor phlorizin through the portal vein, which also suggests the presence of a sodium glucose-linked transporter (or a related protein) in the bile ducts [41, 42]. While the glucose content of bile in horses has not yet been studied, our findings of SGLT1 in the equine bile ducts suggest a similar glucose reabsorptionalthough care was takensystem. A positive SGLT1 antibody signal was also present in Kuppfer cells in all horses, with inter-individual variation in the number of positive cells. The staining was coarse granular and close to the nucleus, possibly suggesting staining of lysosomes or other vesicles [43]. Most studies of SGLT1 protein expression in other species do not describe Kuppfer cells [28, 29, 32], and therefore it is not possible to say if it represents a feature exclusive to horses, or simply an non-specific staining.

The Invitrogen SGLT2 antibody used in our study displayed a positive signal in hepatocytes in a zonal distribution, with increased intensity around the central vein. The zonal distribution of different monosaccharide transporters have been reported previously in mice [44], and might mirror differences in glucose metabolism across different zones of the liver [45]. Reports of hepatocyte SGLT2 protein or *SLC5A2* gene expression in other species are conflicting, with some studies suggesting high presence in humans and mice [23, 26], while others failed to find mRNA in humans or rats [29, 46]. In bovines, low amounts of both SGLT1 and SGLT2 mRNA were found in the liver using Northern blot [47, 48]. In general, there is a lack of published research on the localisation of SGLTs in the liver, and further studies are needed across different species. The emergence of SGLT2i as a successful treatment for Metabolic Dysfunction- Associated Steatotic Liver Disease (MASLD) in humans [49] raises questions about the underlying treatment mechanisms. It is yet unclear whether these are all related to overall increased metabolic health, or due to any direct effect on hepatic SGLT2.

In the present study, neither one of the tested SGLT1 antibodies produced any staining in pancreatic tissue, while a distinct granular intracellular signal was detected in cells in the islets of Langerhans within the pancreas with the use of the Invitrogen SGLT2-antibody. The staining intensity and number of SGLT2-positive cells varied strongly between individuals. In other species, the findings of both SGLT1 and SGLT2 protein in endocrine pancreas is controversial, and studies performed show contrasting results in mice, rats and humans [50]. In human pancreatic islets, SGLT2 protein was found to be co-localised with glucagon, but the authors noted a pronounced intraindividual variation between the number of positive cells in each islet [25]. According to previous studies, the equine pancreatic islets have a unique cellular organisation with α-cells contained centrally with a mantle of β-cells in the periphery; the opposite of the organisation seen in rodents [51–54]. As the staining was mostly contained within cells in the centre of the islets, and in accordance with the studies where SGLT2 antibody signal was mostly co-localized with glucagon-secretion [24, 25], we hypothesize that the positive signal seen in equine islets comes primarily from α-cells. Double-staining with antibody against glucagon is necessary to confirm this result, but was beyond the scope of this study. The variation of SGLT2 staining intensity seen in horses might reflect interindividual variability of the number of different cell types between islets, as seen in humans [25]. Another possibility is that the variation can be explained by regional differences between endocrine cell type prevalence in different parts of the equine pancreas [51], although care was taken to ensure that samples were taken from the same pancreatic region in all horses. In addition, a diffuse intracellular staining was seen in a varying amount of extrainsular pancreatic cells in all horses. These cells might represent endocrine cells within the exocrine pancreas, as previously reported in horses [52]. However, glucagon-secreting cells in the exocrine pancreas were mostly found close to the pancreatic ducts [51], as was not seen in our study. Another option is that these SGLT2-positive cells represent some other cell type in the exocrine pancreas (or a specific stadium of acinar cells) present in varying numbers between individuals.

The role of SGLTs in endocrine pancreas is not fully elucidated. As mentioned in a comprehensive review on the subject, SGLT2i treatment is known to increase endogenous glucose production and plasma glucagon levels in human diabetic subjects [50]. This effect has to our knowledge not been investigated in horses. If present, this could in part explain the known side effect of hypertriglyceridemia seen in horses treated with SGLT2i [17, 55], as increased glucagon secretion leads to gluconeogenesis, glycogenolysis and lipolysis. The mechanism behind this increase in glucagon is not fully known, but studies have investigated the potential function of SGLTs as glucose sensors in α-cells, showing that the presence of SGLT2-inhibitors increase glucagon secretion even during normoglycemic conditions [24, 56]. However, ex vivo experiments on perfused rat, mice and human pancreatic islets and in a perfused rat pancreas showed no direct effect of SGLT2-inhibitor infusion on glucagon secretion, suggesting that the effect must be indirect [57, 58]. As mentioned above, studies differ in findings of SGLT1 and SGLT2 in islet cells, further casting doubts over the exact mechanism by which SGLT2i affect glucagon secretion, and more studies are clearly needed to elucidate the exact mechanisms.

In this study, an incidental histological finding of hemosiderin in liver and pancreas was seen (Supplementary image S6). In liver tissue from five out of ten horses, brown intracellular granules were present in both Kuppfer cells and hepatocytes, as previously reported in donkeys [59]. The cellular localisation and frequency (50%) reported in this study is in accordance with previous findings where 42% of healthy horses had mild to moderate hemosiderin deposits at necropsy [60]. The brown granules found contained within cells in exocrine pancreas likely also represents hemosiderin, as these findings came from the horses with the highest number of hemosiderin-containing cells in the liver. The pancreas is prone to hemosiderin deposits, as shown in a case series of horses with chronic iron overload [61]. However, in the horses of the current study, this likely represents a benign incidental finding.

Due to the lack of commercially available equine-specific SGLT protein antibodies, human specific antibodies were used in the current study. Homology in amino acid sequence between human and equine SGLT1 and SGLT2 is however 88,8% [36] and 92,3% [62], respectively. Antibodies against a similar epitope as the SGLT1 antibody from Fabgennix has been previously used in horses [36, 37]. The SGLT2 antibody from Invitrogen was chosen based on a 100% immunogen sequence homology with equine SGLT2. It has previously been used to evaluate kidneys and pancreas in rats, mice and humans, and while renal SGLT2 protein expression was high in all species, no evidence of pancreatic SGLT2 has been reported [58]. Furthermore, the Invitrogen SGLT2 antibody was used to validate the ultrastructural localisation of the antibody labelling, using an immunogold labelling technique visualised by TEM. The positive signal was clearly localized to the apical membrane in both antibody concentrations tested, and this corresponds with previous findings of SGLTs in epithelia of proximal tubules [63]. The low but clear mitochondrial signal can be an indication of antigen presence (due to SGLT2 also being present in mitochondria, albeit in very low concentration) or a non-specific signal. Cell nucleus staining was not present in low concentrations (while microvilli signal was maintained), indicating that it was non-specific. To further validate our IHC results, a second set of antibodies against different epitopes of human SGLT proteins were used. Renal tissue staining pattern from the Abcam SGLT2 antibody was identical to the Invitrogen SGLT2 antibody, confirming the result (Supplementary image S4A-C). The Invitrogen SGLT1 antibody signal showed a similar pattern to the Fabgennix SGLT1 antibody in renal tissue but with a considerably higher background stain (Supplementary image S5A-C). Additionally, in tissues with lower expression, the results differed more significantly between the original and the validation antibody. The second SGLT1 antibody (Invitrogen) failed to provide a positive signal in cholangiocytes and gave a generally higher background staining present in all cell types, even at low concentrations (Supplementary image S5D-F). Similarly, hepatocyte signal was weaker and the positive signal from cells within the endocrine pancreas was not seen with the validation SGLT2 antibody from Abcam (Supplementary image S5D-I). This might reflect the lower specificity of these antibodies towards equine glucose transporters, which may lead to lower signal in tissue with low protein expression, echoing findings of a murine study testing multiple commercial SGLT1 antibodies simultaneously using SGLT1-knock out mice as negative control [28]. Ideally, the immunohistochemistry would have been performed with antibodies specifically raised against equine glucose transporters, but this was beyond the practical and economical scope of our study. Western blot was also considered as a validatory step, but was not performed based on low availability of suitable antibodies that could be used for this technique. In addition, for three out of four chosen antibodies, no commercial blocking peptide was available, limiting our options to perform tests of non-specific binding in studied tissues.

A limitation of our study is the study population consisting of one breed only, which was a result of sampling method (convenience sampling). Furthermore, although individuals with signs of systemic illness were excluded, the lack of a thorough medical history and clinical examination prior to euthanasia may have led to inclusion of individuals with diseases not easily detected at necropsy, possibly affecting protein expression - for example EMS or pituitary pars intermedia dysfunction (PPID). However, as the study was qualitative in nature and findings were very similar across individuals (save in the pancreas, see discussion above) any negative effect of this was not evident.

Additional studies on the effect of disease or treatment on SGLT1 and SGLT2 expression in equine tissues are needed, as changes might indicate the need for dose adjustments in long-time treatment of EMS with SGLT2i. Studies in other species show different results depending on disease models, as a study in multiple metabolic disturbances in rats demonstrate [64]. Therefore, quantitative studies on protein expression in equine kidneys need to be performed on treated *versus* un-treated horses with EMS, to assess whether SGLT2-inhibitor dose should be adjusted over time.

## Conclusion

The present study describes presence and localization of SGLT2 and SGLT1 in the equine kidney, liver and pancreas. Our results indicate that SGLT2 is the predominant glucose transporter in the equine kidney, located in the brush border of the epithelial cells of the proximal convoluted tubules. The subcellular localisation was confirmed with ultrastructural imaging. Our results support that the proposed mechanism of SGLT2i treatment in equines is similar to results shown in laboratory animals; SGLT2-inhibitors prevent glucose reabsorption by tubular epithelium in the proximal convoluted tubules, leaving SGLT1 as the one remaining available glucose transporter. This leads to an increase in urinary glucose excretion, a lowering of systemic glucose load and can thereby decrease the insulin response. Extrarenal protein expression of SGLT2 in the hepatocytes and in pancreatic islets, and of SGLT1 in the cholangiocytes may reflect other mechanisms by which the desired treatment effect is achieved, or play a role in the side effect profile. Further research is needed into the roles of these extrarenal SGLT2 and SGLT1 in equines, and into the change of renal SGLT protein expression levels during long time treatment and disease.

## Supplementary Information

Supplementary Material 1.

## Data Availability

Original data supporting the findings of this study are available from the corresponding author upon reasonable request.

## Abbreviations

SGLT2i: Sodium–glucose linked transporter 2 inhibitor
SGLT1: Sodium–glucose linked transporter 1
SGLT2: Sodium–glucose linked transporter 2
EMS: Equine metabolic syndrome
ID: Insulin dysregulation
IHC: Immunohistochemistry
TEM: Transmission electron microscopy
