# Supplementary material for: Sodium-glucose transporters SGLT1 and SGLT2 in equine renal, hepatic and pancreatic tissue
Source: BMC Vet Res. 2025 Dec 17;22:36. doi: 10.1186/s12917-025-05173-1 (PMC12822031; doi:10.1186/s12917-025-05173-1)
Supplement: Supplementary file 1 — Supplementary Material 1. [file 12917_2025_5173_MOESM1_ESM.pdf]

**Supplementary Table 1**

| CASE | BREED | AGE | WEIGHT | SEX | LACTATION |
|---|---|---|---|---|---|
| 1 | NCBT | 16 | 565 | Mare | No |
| 2 | NCBT | 3 | 545 | Gelding | - |
| 3 | NCBT | 4 | 485 | Stallion | - |
| 4 | NCBT | 5 | 485 | Mare | No |
| 5 | NCBT | 16 | 460 | Mare | Yes |
| 6 | NCBT | 4 | 475 | Mare | No |
| 7 | NCBT | 4 | 580 | Gelding | - |
| 8 | NCBT | 10 | 510 | Stallion | - |
| 9 | NCBT | 16 | 470 | Mare | Yes |
| 10 | NCBT | 3 | 560 | Mare | No |

Supplementary table 1: Information about breed, age, weight, sex and lactation status of horses used in study. NSCT = Norwegian/Swedish Coldblood Trotter. Age in years. Slaughtered weight in kg.

## Supplementary Table 2

| Target | Manufacturer | ID | Immunogen | % identity eSGLT1 | %identity eSGLT2 | aa sequence |
|---|---|---|---|---|---|---|
| SGLT1 | Fabgennix | SGLT-101AP | aa 402-422 (1a) | 90% (1b) | 65% (1a) | STLFTMDIYAKVRKRASEKEL |
| Host/isotype: | | Polyclonal rabbit IgG anti-human | | | | |
| SGLT1 | Invitrogen | PA5-84237 | aa 224-261 (2a) | 79% (2b) | 32% (2b) | EVGGYDAFMEKYMKAIPTIVSDGNTTFQEKCYTPRAD |
| Host/isotype: | | Polyclonal rabbit IgG anti-human | | | | |
| SGLT2 | Invitrogen | PA5-34210 | 17 aa, C-terminus (3a) | 100% (3b) | <36% (3b) | unknown |
| Host/isotype: | | Polyclonal rabbit IgG anti-human | | | | |
| SGLT2 | Abcam | ab85626 | within aa 250–350 (4a) | 87% (4b) | 60% (4b) | unknown |
| Host, isotype: | | Polyclonal rabbit IgG anti-human | | | | |

Supplementary table 2: Protein target, manufacturer, ID, immunogen and calculated homology of target antibody in commercial primary antibodies used in study. For a detection of primary antibodies, a universal secondary horseradish peroxidase-conjugated antibody (MACH1 Universal HRP-polymer, MRH538, Biocare Medical) was used in all experiments

Source 1, a: Thermofisher homepage, b: BLAST analysis uniprot.org

Source 2, a: Thermofisher homepage, b: BLAST analysis uniprot.org

Source 3, a: Thermofisher homepage, b: BLAST analysis uniprot.org

Source 4, a: personal communication Abcam, b: Chiba *et al,* 2020

***Sources:***

Chiba Y, Murakami R, Matsumoto K, Wakamatsu K, Nonaka W, Uemura N, Yanase K, Kamada M, Ueno M. Glucose, Fructose, and Urate Transporters in the Choroid Plexus Epithelium. Int J Mol Sci. 2020 Sep 30;21(19):7230. doi: 10.3390/ijms21197230. PMID: 33008107; PMCID: PMC7582461.

Consortium TU. UniProt: the Universal Protein Knowledgebase in 2025. Nucleic Acids Research. 2025;53:D609–D17

**Supplementary Image S1**

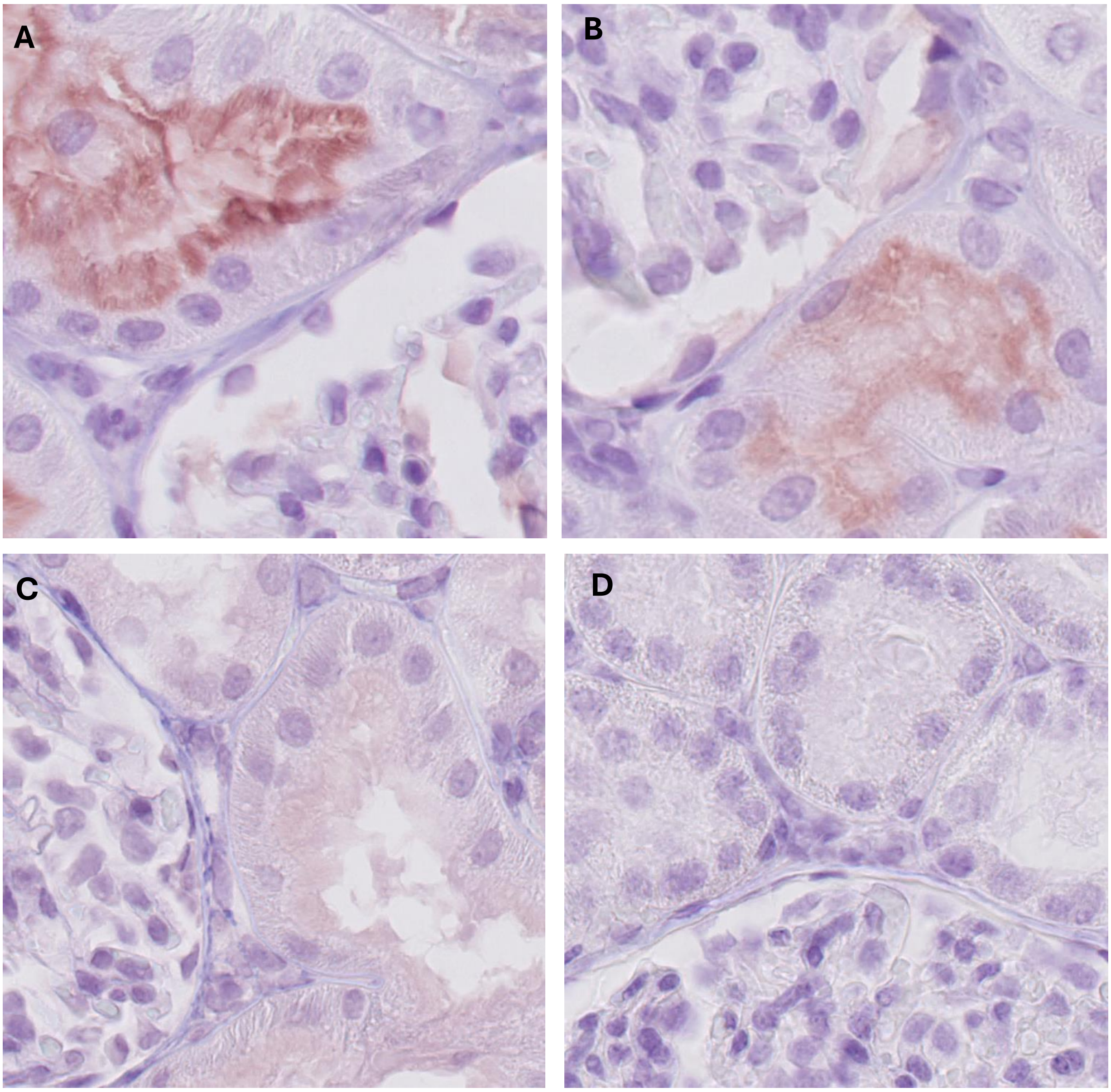


S1: Example of strong (A), mid (B), weak (C) or no (D) apical staining. Light microscopy of equine renal tissue stained with SGLT2 antibody (A, B), SGLT1 antibody (C) or no primary antibody (D).

**Supplementary Image S2**

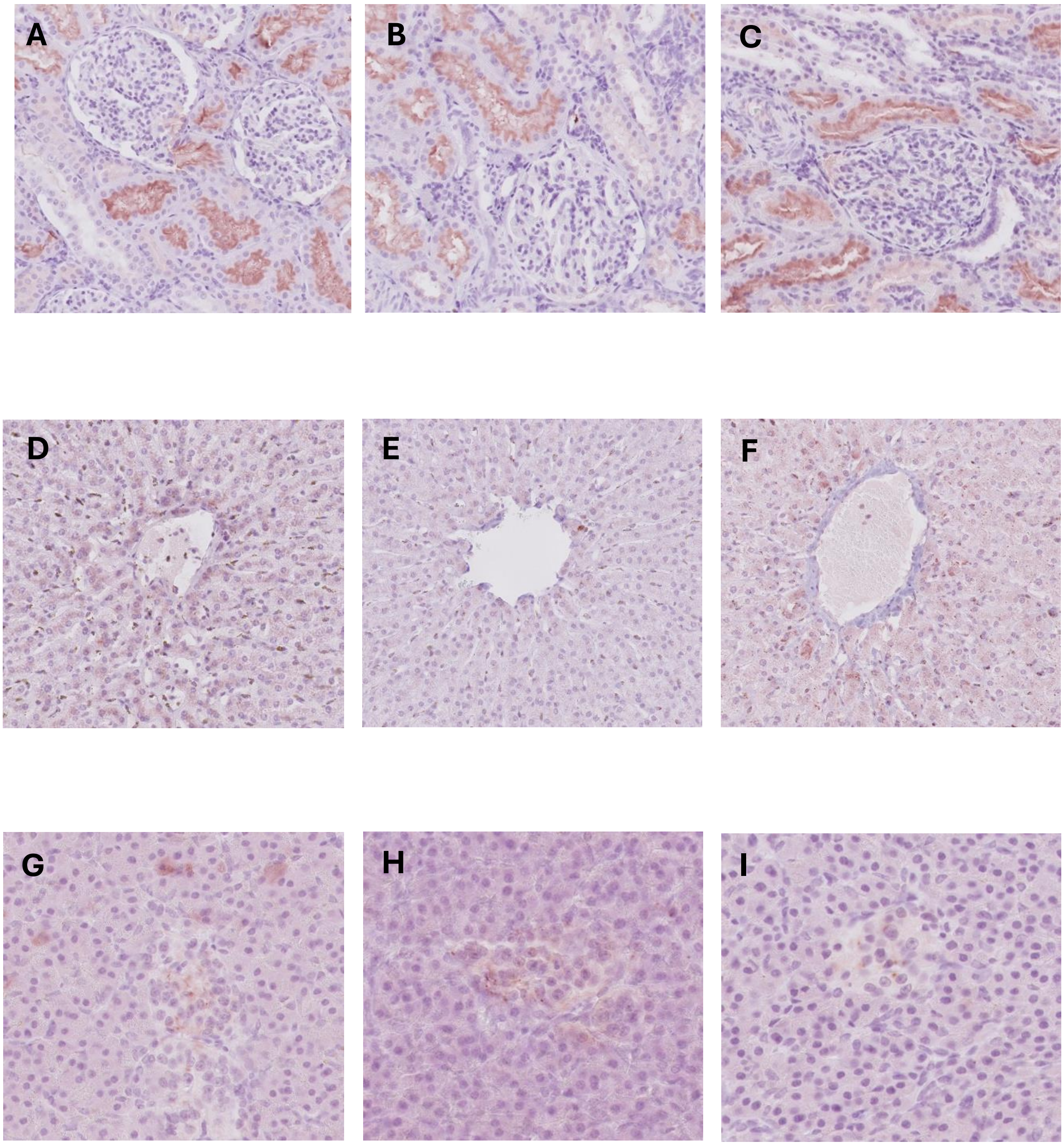


S2: Light microscopy of renal (A -C), hepatic (D-F) and pancreatic tissue (G-I) from different horses stained with SGLT2 antibody (Invitrogen), in concentration 1:1000.

**Supplementary Image S3**

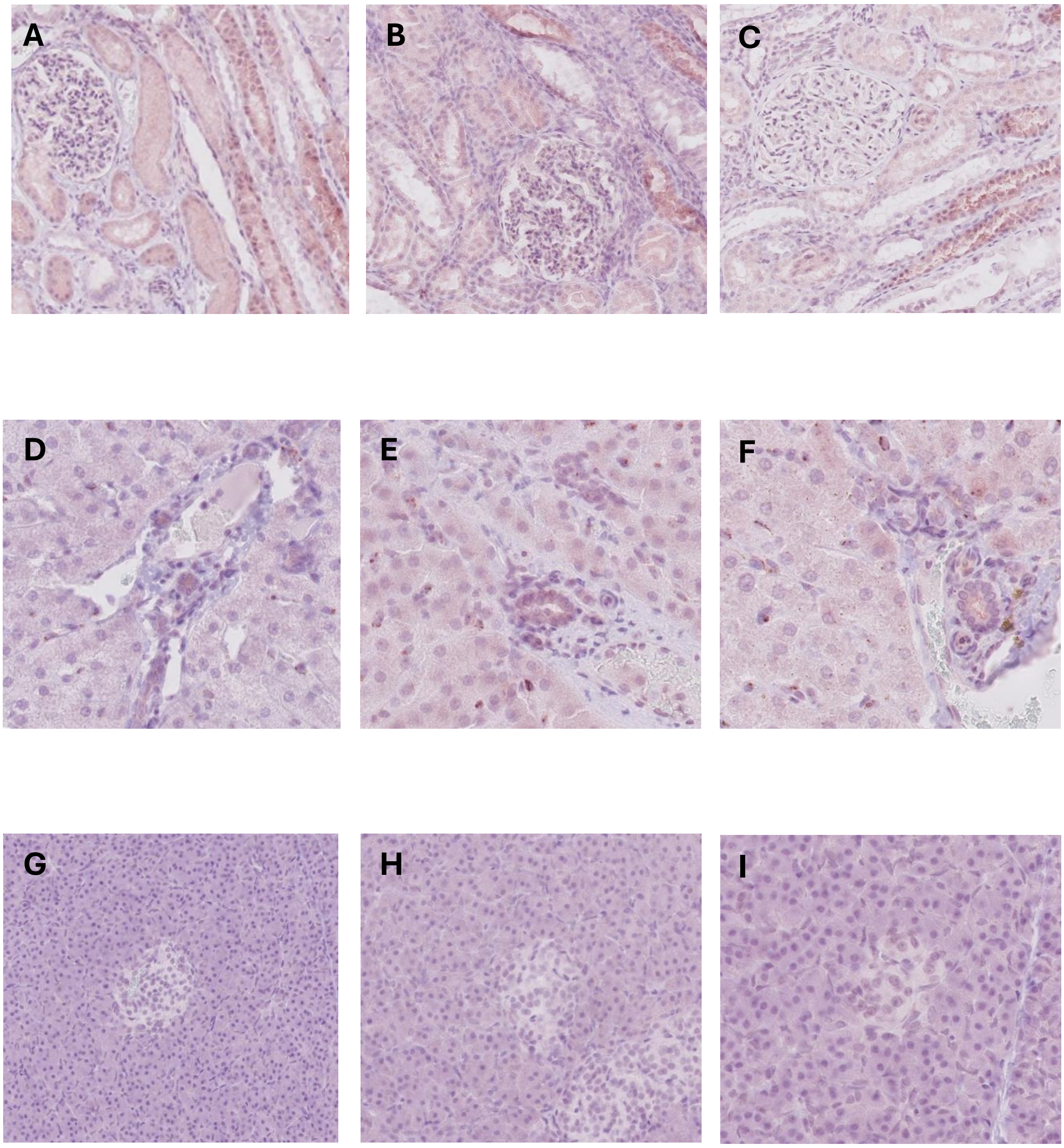


S3: Light microscopy of renal (A -C), hepatic (D-F) and pancreatic tissue (G-I) from different horses stained with SGLT1 antibody (Fabgennix), in concentration 1:1500.

## Supplementary Image S4

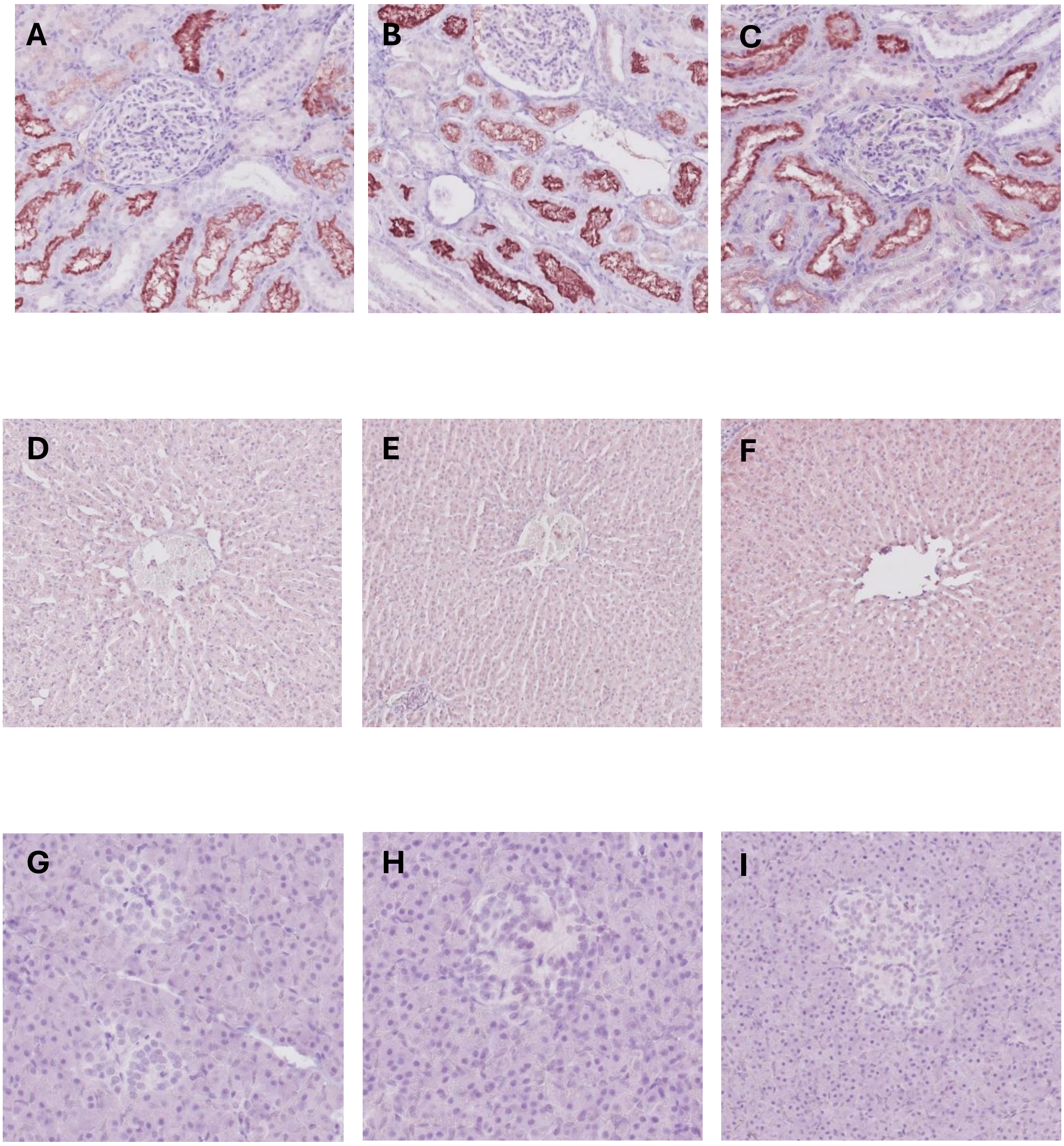


S4: Light microscopy of renal (A -C), hepatic (D-F) and pancreatic tissue (G-I) from different horses stained with SGLT2 antibody (Abcam), in concentration 1:10000.

**Supplementary Image S5**

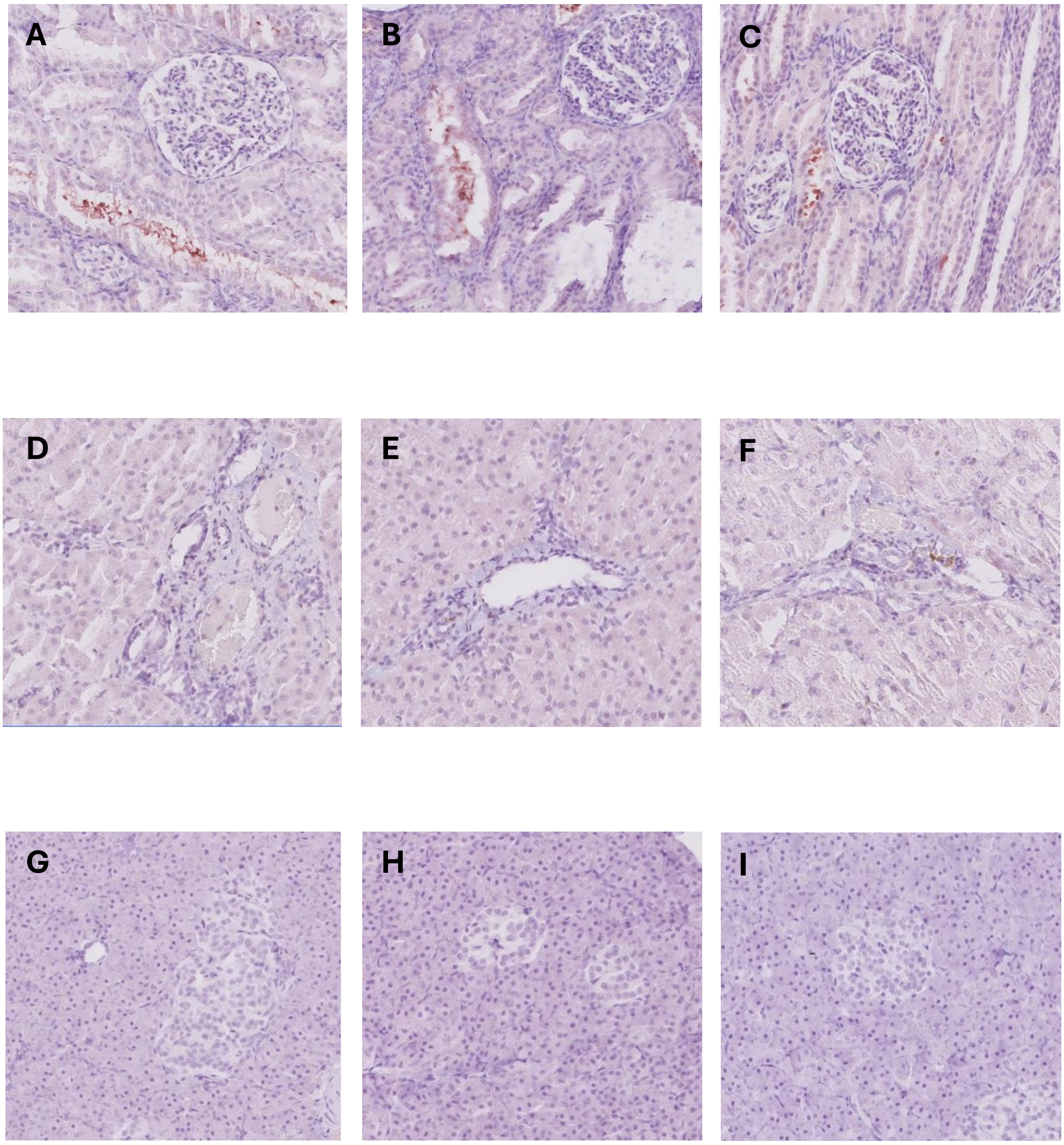


S5: Light microscopy of renal (A -C), hepatic (D-F) and pancreatic tissue (G-I) from different horses stained with SGLT2 antibody (Invitrogen), in concentration 1:5000.

**Supplementary Image S6**

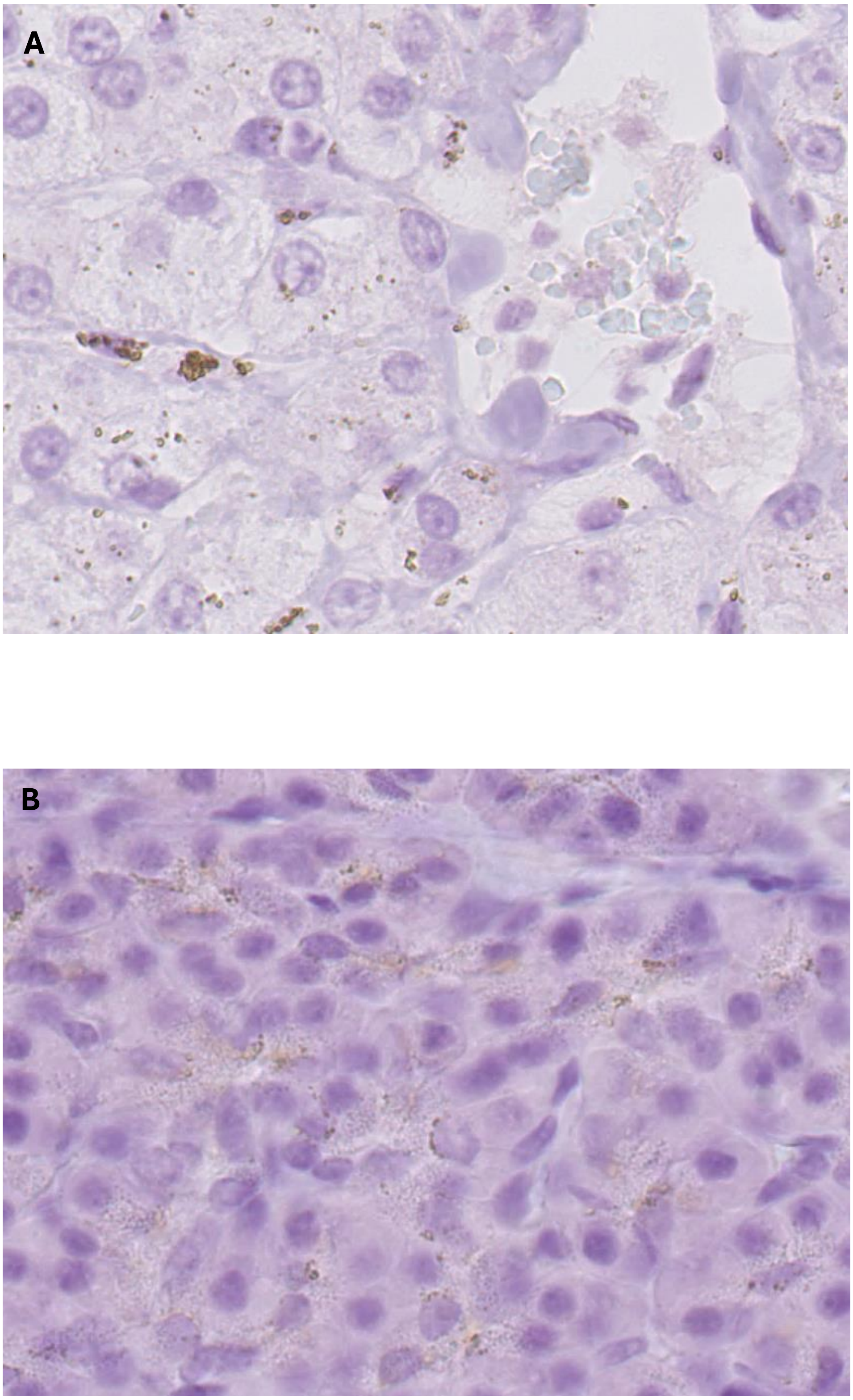


S6: hemosiderin deposits in negative controls (no primary antibody used) in A) equine liver B) equine pancreas, exocrine tissue.
